# Biochemical laboratory management with a microcomputer

**DOI:** 10.1155/S146392468600041X

**Published:** 1986

**Authors:** R. Kowalezyk, G. Morgant, F. Ch. Baumann, J. Giboudeau

**Affiliations:** 1 Laboratoire de Biochimie A Hôpital Saint Antoine 184, rue du Fg Saint Antoine Paris Cedex 12 F 75571 France


**Journal of Automatic Chemistry**/*Journal of Clinical Laboratory Automation*, **Vol. 8, No. 4 (October–December 1986), pp. 211–214**

### Biochemical laboratory management with a microcomputer

**R. Kowalezyk, G. Morgant, F. Ch. Baumann and J. Giboudeau**

*Laboratoire de Biochimie A, Hôpital Saint Antoine, 184, rue du Fg Saint Antoine, F 75571 Paris Cedex 12, France*

### Introduction

The very large number of analyses carried out in the authors' laboratory (2500 to 3500 per day) inevitably causes a backlog in administrative work (copying, archiving, activity register etc.). In order to reduce this time-consuming activity which can lead to errors, it was decided to replace a non-operational minicomputer (Digital PDP 11/03) with a new generation microcomputer.

The change was made within stringent financial limits and within the requirement of expanding the system to the whole laboratory. A multi-user computer with a large capacity for expansion was needed; initially, one analyser was linked to the system (500 to 800 analyses per day), two others are now connected (1000 to 1500 per day), and a fourth will follow very shortly (500 to 1000 per day).

### Hardware, operating system and development software

The Micromega 32:16 (Alcatel Thomson) (or Fortune 32:16) is a powerful microcomputer with a MC 68000 microprocessor, a large main memory (1 Mbytes) and one to four fixed disk drives (see table 1).

The MIMOS operating is a French version of the UNIX system III (Bell Laboratories) including some features of the Berkeley 2·8 and 4·1. This operating system is fully adapted to multi-user operation.

The first development software was the SMC Business BASIC (Alcatel Thomson), which is particularly well-adapted to management applications. The language chosen for further development is 'C' giving high running-speed programs.

### Tailor-made software (C.I.B. 10, boulevard Malesherbes, F 75008 Paris, France)

### *Requirements and initial solutions*

The authors' laboratory is divided into several units (see figure 1), which are completely independent of each other

*Table 1. Specifications.*

| | | |
|---|---|---|
| *Computer* | | |
| Microprocessor | : | MC 68000 MOTOROLA/THOMSON-EFCIS |
| Clock frequency | : | 5·5 MHz |
| Main memory | : | 1024 Kbytes (512 to 2048 Kbytes) |
| Floppy disk drive | : | 5·25-inch 770 Kb formatted |
| Fixed disk drive | : | 5·25-inch 70 Mb Winchester disk drive |
| In–out lines | : | 1 asynchronous RC 232 C port on mother board |
| | | 2 four-channel asynchronous serial-line controllers |
| *Terminals* | | |
| Video display | : | 12 in. green (P39 phosphorus) |
| Display capacity | : | 25 lines of 80 characters |
| Keyboard | : | detachable, standard, functions and numeric keypad |
| Communication | : | RS 232 C line |
| *Printer* | | |
| Manufacturer | : | Data Recording Equipment (UK) |
| Model | : | 8840 |
| Printing | : | Impact dot matrix |
| | | Bidirectional with head motion optimization |
| Print speed | : | 240 cps or 160 lines per minute at 80 characters per line |
| Interface | : | Standard RS 232 C |

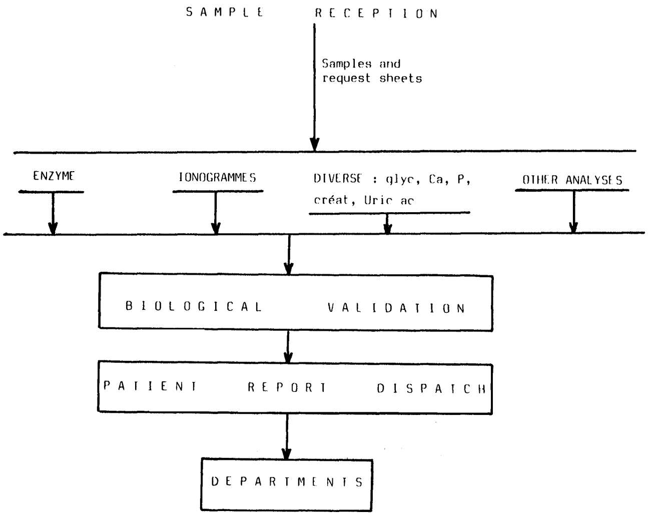

*Figure 1. Overall laboratory organization.*

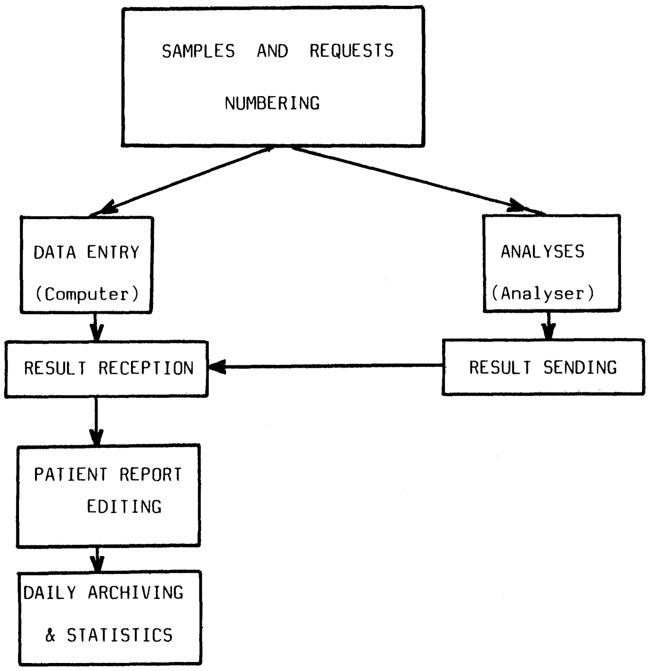

*Figure 2. Unit organization.*

in order to give maximum flexibility and rapidity in the execution of the analyses and to allow for possible equipment or computer failure. Therefore the following were chosen for the project:

(1) Tailor-made software.

(2) The program was designed to be run in three independent parts (one for each laboratory unit) but with the same overall architecture (figures 2 and 3).

(3) The analysers were linked in unidirectional mode to ensure independent analyses and data-processing.

(4) Conversation with users was designed to be as simple as possible and self-documented input screens were supplied to remove the need to refer to manuals.

(5) There was no computer check of data validity—it was considered that the integrated checks carried out by the analysers, plus those of the technicians, would be sufficient to ensure the validity of the results.

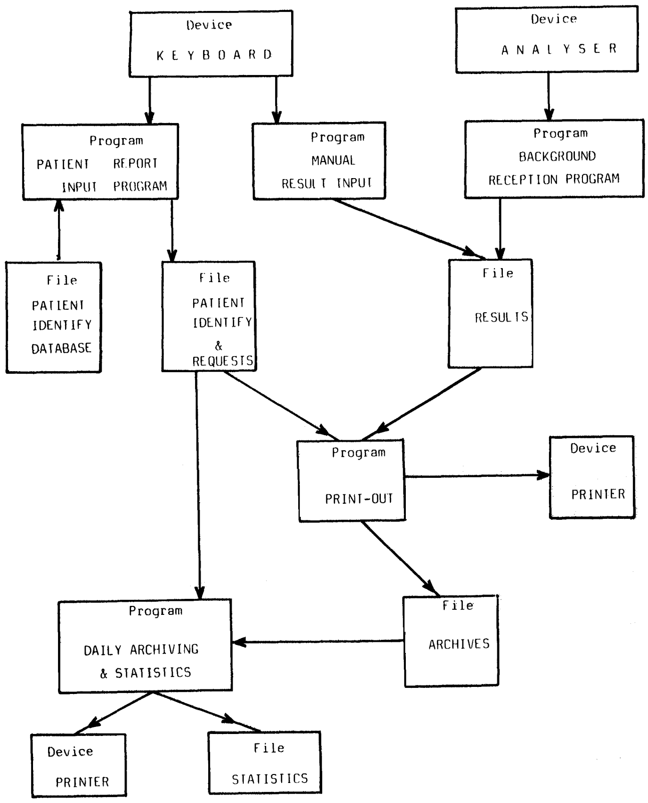

*Figure 3. Software structure.*

### Software function

*Connection of analysers*

The three analysers in use in the authors' laboratory (Greiner G 300, Beckman Astra 4 and 8) possess standard RS 232 C serial interfaces, which make connection to a computer relatively easy. However, some problems were encountered:

(1) The total lack of hardware handshaking and the high speed of output (9600 bauds).

(2) Continuous sending of characters' blocks, for example the GREINER G 300 sends five-character blocks every 2 s and a result blocks (56 characters) every 12 or 24 s in full operation. The ASTRA 4 and 8 send result blocks (variable length) every 60 s, but possess a special function which allows result recall. Output speed, however, is then limited by the internal thermal printer.

These problems are solved with the UNIX operating system by setting 'I/O drivers' to interrupt the processor for each character received (thus the lack of handshaking is quickly solved); by background running of process (process without terminal communication); and by the UNIX nucleus assuming dynamic process management. Thus an input process which does not receive data is set in a dormant state and does not affect system performance. The presence of one or more background input waiting processes does not change the system's response time for other processes (such as terminals).

*Table 2.*

HOPITAL SAINT-ANTOINE

LABORATOIRE DE BIOCHIMIE A

| | |
|---|---|
| NOM........... | SERVICE......... CATTAN |
| PRENOM........ | IDENTIFICATION.. 29491 |
| REMARQUE : | DATE : 1983/12/19 & HEURE : 16H52 |

BILAN SANGUIN

| ANALYSES | RESULTATS-UNITES | | VALEURS USUELLES DU LABORATOIRE |
|---|---|---|---|
| CALCIUM ...... | 2.25 | millimol/l | H 2.25 -2.52 & F 2.17 - 2.46 |
| PHOSPHORE.... | 1.12 | millimol/l | H 0.76 - 1.16 & F 0.83 - 1.26 |
| CREATININE... | 80 | micromol/l | H 79 - 111 & F 60 - 94 |
| AC. URIQUE... | 545 | micromol/l | H 276 - 428 & F 170 - 333 |
| UREE....... | 5.2 | millimol/l | 3.4 - 7.1 |
| FER.......... | 25.4 | micromol/l | H 10.7 - 26.9 & F 8.3 - 24 |
| CAP. FIXATION | 45.2 | micromol/l | H 48.6 - 67.2 & F 49.4 - 73.1 |
| SATURATION... | 56.2 | p. cent | |
| GLYCEMIE(S) | | | |
| A JEUN.......... | 5.2 | millimol/l | 4.1 -> 5.8 |
| POST-PRANDIALE.. | 6.8 | millimol/l | |
| cf COMMENTAIRE. | 8.5 | millimol/l | |

COMMENTAIRE : CHARGE GLUCOSE 706

BILAN URINAIRE

DIURESE= 1500 ml

| | | | | | |
|---|---|---|---|---|---|
| CALCIUM...... | 2 | millimol/l | soit | 3 | millimol/24h |
| PHOSPHORE.... | 2.5 | millimol/l | soit | 3.75 | millimol/24h |
| CREATININE... | 4.5 | millimol/l | soit | 6.75 | millimol/24h |

CLAIRANCE(S)

| | | | |
|---|---|---|---|
| CREATININE... | 0.980 | ml/sec | |
| PHOSPHORE.... | 0.040 | ml/sec | Taux reabsorption 96 p. cent N 84-91 |

LE CHEF DE LABORATOIRE

Some problems can occur due to the multiplication of these waiting processes because of the high transmission speed and the possible appearance of characters on several lines. The interrupt management system then chooses on line and some data can be lost. At 9600 bauds, three processes seem to be the maximum.

In order to expand the system, the data reception principle has to be changed and each analyser linked through a buffer memory. The three programs, additionally, have to be replaced by one which checks each channel in turn for the arrival of data. This system allows for expansion to 10 analysers (limited by the number of I/O lines of the computer).

The operation of the data-processing programs is very simple: suppress unwanted information, characters or block, extract analyses number, result, date and time, different codes (error, units etc.) and store of the fixed disk.

### Global organization

The technician's work can be divided into the following steps:

(1) Receiving samples and requests.

(2) Numbering.

(3) Analyses and entering requests.

(4) Checking results and repeating analyses if necessary.

(5) Printing result sheets.

(6) Archiving.

### Entering patient reports

Each report includes the following features: *identity:* identification number (optional), name, surname, department, clinical data; and *item:* requested analyses, diuresis, comments.

Given the large number of analyses, for each unit (up to 300), it was important to accelerate this step. The following solutions were chosen: use of patient admission number (when available) for direct patient identity recall from a file; the possibility of entering incomplete identity (e.g. poorly written); total independence between reports opened on the same day for the same patient; and use of panels, either for one or for a series of reports.

### Acquisition of results

The acquisition of results is made through connection programs but can also be carried out manually (checking, manual analyses etc).

### Editing

Several types of print-out can be obtained and fall into two categories: usual: result sheets, archives and statistics; unusual: list of results received, work-list, list of departments.

### *Result sheets (table 2)*

Each unit requests the generation of its result sheets in different ways: singly or in series one or several copies.

Each report includes laboratory identification, sequential day number, results, units, usual laboratory range. The printing program also includes checking between the requests and the results recevied: if an error occurs, the report printing is skipped and a message is printed on the screen.

### *Daily archiving*

To prevent data loss, paper archives are generated each day.

This is divided into two steps: firstly, checking that all reports have been printed; and secondly, an alphabetical print-out of the result-sheets in short format (three or four lines by sheet). At present this step is independent for each of the three units, but will shortly be grouped.

### *Accounting*

To obtain detailed information on laboratory activity, the system accounts all analyses (daily and by parameters). The results are printed-out daily and stored on fixed disk for monthly accounts tables.

*File up-date*

After all the previous operations have been successfully carried out the files are updated in the following way: archive file is initiated, report and result files are transferred on the FIFO principle (first-in, first-out).

*On-line report call-up*

It is very easy to call-up a current or a past report using few letters (from one to four) of the name. Storage time depends on the fixed disk capacity; in the authors' laboratory it has been fixed at 30 days (which is approximately 10 Mbytes).

### Discussion

The initial requirements of the system (fast data handling, flexibility) have been fully met. Given the relatively limited capacity of the computer, and the large number of analyses carried out in the laboratory, performance was improved by the following means: rapid report acquisition through simplified input, unit independence, unidirectional connection of analysers and manual data validation. However, some criticisms can be made: as the reports are generated independently, results for the same patient from different units and different days cannot be grouped (it will, however, soon be possible to group same-day reports during the archiving and call-up).

Additionally, the system is very reliable: only one breakdown (a fixed disk failure) has occurred during 24 months' continuous operation for the computer and the printer has suffered one power failure. The software is user-friendly and is operated by a variety of staff (medical, technical, administrative etc.). The system is clearly low cost in relation to performance and possibilities of expansion.

Overall, the system is very well-suited to the structure of the authors' laboratory and to the hospital, which has 108 intensive-care beds, a rapid bidirectional flow of information is extremely important and a project is now underway to set up bidirectional connections with intensive-care units.

The next version of the software will be partially in 'C' language for faster running and more available memory. It will also include such advanced functions as result checks, automatic validation, grouping of reports and automatic background file up-date.

### Technicon RA users' meeting

A meeting for users of Technicon RA systems was recently held at Asburne Hall, University of Manchester. Chaired by Dr Toothill from Old Medical School, Leeds, 100 delegates listened to presentations by fellow users and Technicon personnel. The wide range of topics discussed included aspects of quality control, applications of various assays to the systems together with papers concerning the adaptation of the RA-100 to different laboratory situations. A poster session was also presented by users.

Several new diagnostic kits were introduced by Technicon, the most significant being a new assay for serum iron plus a simple latex agglutination/inhibition assay for measurement of theophylline. Further details plus abstracts of the papers presented can be obtained on request.

This was the first meeting for RA users to be held over two days and, due to its obvious success according to comments from delegates, will almost certainly be the format for such future events.

*More information from Francis Hooley, Technicon on 0256 29181.*

